# Crhr1 and epinephrine utilize the central Ras-MAPK pathway in mediating the acute stress-related locomotor activity in zebrafish larvae

**DOI:** 10.3389/fendo.2025.1650458

**Published:** 2025-09-11

**Authors:** Enezi Khalid, Mathilakath M. Vijayan

**Affiliations:** Department of Biological Sciences, University of Calgary, Calgary, AB, Canada

**Keywords:** cortisol, stress response, behavior, HPI, CRH, c-Fos, fight-or-flight response

## Abstract

**Introduction:**

Although the Crh-Crhr1 system is the proximal trigger for the stressor-induced corticosteroid release, its role in initiating the fight-or-flight response to an acute stressor is unclear. We hypothesized that the Crh-Crhr1 system deploys the central Ras-Mapk (mitogen-activated protein kinase) pathway and rapidly increases the locomotor activity in zebrafish larvae.

**Methods:**

We tested this using an acute stressor-induced hyperactivity model in larval zebrafish that is Crhr1-dependent, and a pharmacological inhibitor of Ras (BAY-293).

**Results:**

The larval hyperactivity response to stress disappeared after pretreatment with BAY-293. Acute CRH exposure stimulated the hyperactivity but at a lower magnitude than epinephrine; however, both responses were inhibited by BAY-293. Immunohistochemical localization revealed rapid phosphorylation of ERK1/2 in the pallium and hypothalamic regions after acute CRH and epinephrine treatment. The lack of Crhr1 (*crhr1-/-*) upregulated the a1-adrenoceptors (*adra1ab* and *adra1ba*) and abolished the epinephrine-induced, but not the forskolin-induced hyperactivity. The acute stressor also increased the transcript abundance of *c-fos*, commonly used as a marker of neuronal activation and plasticity. This immediate early gene response to stress was mimicked by epinephrine, but not Crh treatment, and was Ras-dependent. The acute stressor- or epinephrine-induced *c-fos* response was unaltered in larvae lacking a functional Crhr1.

**Discussion:**

This study reveals the activation of the Ras-Mapk pathway by Crhr1 as a central mechanism modulating the acute stress-induced larval hyper-locomotor activity but not the c-fos response in zebrafish. Altogether, our results suggest a complementary but essential role for Crhr1 in facilitating the epinephrine-mediated fight-or-flight response but not the stress-habituation response.

## Introduction

In vertebrates, detection of a real or perceived stressor leads to a complex neurobiological response that is highly conserved to deal or cope with the stressor (1). In teleosts, as in other vertebrates, the whole-body stress responses are initiated upon stressor perception by two major arms, the sympathetic nervous system (SNS) and the hypothalamus-pituitary neuro-endocrine stress axis (2, 3). Chromaffin cells in the interrenal tissue are activated through the brain sympathetic pathways to stimulate catecholamine release, which mediate rapid actions on the cardiovascular and musculoskeletal systems to facilitate the fight-or-flight response to acute stress (4). In parallel, the canonical hypothalamus-pituitary-interrenal (HPI) axis, which is analogous to the HPA (adrenal) axis in mammals, is initiated by the release of corticotropin-releasing hormone (Crh) from neurons in the neurosecretory preoptic area (5, 6). The Crh activates the Crh receptor 1 (Crhr1) in the pituitary, which is the primary receptor involved in the downstream release of cortisol from the interrenal tissues during stress (7–9). Briefly, Crh stimulates the pituitary to release adrenocorticotropic hormone (Acth), which in turn activates melanocortin 2 receptor in the interrenal steroidogenic cells to stimulate the biosynthesis of cortisol, the principal corticosteroid in teleosts (2, 10, 11). Cortisol, through its actions by activating the glucocorticoid receptor (Gr) and the mineralocorticoid receptor (Mr), enacts various physiological changes, including altering metabolism, osmoregulation, musculoskeletal function, as well as modulating various growth, immune, and reproductive parameters (11, 12).

Prior research from our lab has demonstrated a clear temporal distinction in the requirements for various components of the HPI axis in terms of supporting the ongoing behavioral response to stress. For instance, the Crh-Crhr1 system is necessary for initiation of the acute hyper locomotory response (within minutes), whereas the corticosteroid receptors (Gr and Mr) are required for the longer-term (hours) sustenance of the behavioral response (8). However, while there is considerable knowledge regarding the mechanisms underlying corticosteroid actions, especially mediated by long-term transcriptional processes (11, 13), comparatively less is known about the role of the HPI axis for the changes underlying rapid responses to stress, especially in teleosts. In particular, the early fight-or-flight responses are usually associated with the SNS activation, while a mechanistic role for HPI axis neuropeptides in facilitating the early rapid response, independent of longer-term cortisol action, is unknown.

To address this gap in knowledge, we utilized an acute stressor-induced hyperactivity behavioral model that was previously characterized to be regulated by Crhr1 activation in zebrafish (*Danio rerio*) larvae (8, 14). We utilized this behavioral paradigm in the presence of inhibitors of specific signaling pathways to better understand the intracellular stress signaling mechanisms facilitating the rapid stress response. In particular, the mitogen-activated protein kinase (MAPK) cascade is a key rapid intracellular signaling module integrating a vast number of diverse stimuli initiated by cell-surface receptors (15), including adrenoceptors by SNS stimulation, to drive the fight-or-fight response (16–19). MAPKs are classically organized within three-tiered protein kinase cascades, where they are phosphorylated and activated by upstream MAPKKs, which in turn are activated by MAPKKKs. While several MAPK isoforms exist, the best characterized is the ERK1/2 (extracellular signal-regulated kinase), which is activated by its upstream kinase MEK (MAPKK equivalent), which in turn is activated by Raf kinase (MAPKKK equivalent) (15). Through adaptors such as Ras GTPase, the Raf-MEK-ERK cascade can be linked to several growth factor receptors, G protein coupled receptors (GPCRs), as well as integrins and cytokine receptors, encompassing a broad repertoire of extracellular stimuli, including neurotransmitters and hormones (20, 21).

Indeed, during acute stressor exposure, the ERK pathway is a target for catecholamine-mediated rapid fight-or-flight response (22–24), as well as in stress-associated learning and memory (25–29). The MAPKs bring about rapid cellular changes through downstream phosphorylation of cytosolic targets, while also initiating transcriptional responses following its nuclear translocation (15). Among the many transcriptional targets of MAPK is the immediate early gene (IEG) *c-fos*, which serves as a reliable marker of neuronal activation (30, 31). The protein product Fos is itself a component of the AP-1 complex which acts as a transcription factor regulating the expression of a diverse set of genes (32, 33). Interestingly, *c-fos* induction is associated with habituation to repeated stressor, making it a useful molecular marker to monitor neural correlates of acute stress (34–36).

While the molecular mechanisms underlying the SNS-driven fight or flight response are well-defined, less is known about the role of Crh in this acute stress response, especially in teleost models. Evidence exists for Crhr1 activation of ERK in mammalian systems (37) but whether this is conserved across vertebrates is unknown. Also, whether Crh modulates adrenoceptor signaling during acute stress responses *in vivo*, independent of changes in cortisol production, is an important question to consider within the broader framework of the neurobiology of stress. Thus, we investigated the interplay between Crh and epinephrine in modulating the acute stress-induced locomotory activity using the wildtype (WT) and *crhr1^-/-^* zebrafish, used previously as an *in vivo* model of acute stress-related behavior (8, 14). We used pharmacological tools including receptor antagonists and a Ras inhibitor to tease out the stress sensing and signaling pathways activated by epinephrine and Crh. We show that both arms of the central stress sensing system, the SNS and the HPI axis, initiates the acute stressor-induced locomotor hyperactivity and is dependent upon active Ras-Mapk signaling.

## Materials and methods

### Animal husbandry and embryo maintenance

All animal procedures in this study were approved by the University of Calgary Institutional Animal Care Committee (protocol: AC21-0061), as per the guidelines set by the Canadian Council of Animal Care. This study used Tupfel long fin zebrafish (wildtype; WT) and zebrafish lacking a functional Crhr1 (*crhr1^-/-^*) as described previously (8). Adults were maintained in a recirculating system (Techniplast, Italy) on a 14:10 h light:dark cycle, with lights switched off between 2300-0900. Water parameters were maintained as follows: 28°C temperature, pH of 7.5, and 750 μS conductivity. Fish were fed twice daily, with a morning feed of dry pellet food (Gemma micro-500, Skretting, Canada) and an evening feed of live brine shrimp (Artemia; San Francisco Bay Brand, Inc.). Adult breeder fish (typically 6–12 months old) were set up for breeding on a weekly schedule. Following evening feeding, fish were placed into breeding traps along with plastic aquatic plants for environmental enrichment. Embryos were then collected the following morning within 30–60 min after the lights are on. Embryo media (E3; 5 mM NaCl, 0.33 mM CaCl_2_, 0.33 mM MgSO_4_, 0.17 mM KCl, and 0.1 ppm methylene blue) was used to wash the embryos and then plate them in polystyrene Petri dishes (VWR, USA). Embryos were maintained in an incubator with atmospheric air exchange, with temperature set to 28°C and 14L:10D photoperiod. E3 media was changed daily, along with removal of any dead embryos and larvae up until 4 days post-fertilization (dpf), when larvae were used for the experiments. Tricaine methane sulfonate (Sigma, St. Louis, MO, USA) was used at 4g/L for euthanasia of larvae following experiments.

### Reagents

Cortisol (hydrocortisone), Acth, human Crh, epinephrine bitartrate, the Ras inhibitor BAY-293 ((*R*)-6,7-Dimethoxy-2-methyl-*N*-[1-[4-[2-[(methylamino)methyl]phenyl]thiophene-2-yl]ethyl]quinazolin-4-amine), NAN-190 (5HT_1A_ antagonist), phentolamine, propranolol, and haloperidol were purchased from Sigma (St. Louis, MO, USA). Antibodies against phospho-ERK (#4370; RRID: AB_2315112) and total-ERK (#4695; RRID: AB_390779) were purchased from Cell Signaling Technology (Danvers, MA, USA). β-actin-Cy3 conjugated monoclonal antibody was purchased from Sigma (C5838; RRID: AB_258912).

### Stress-related behavioral analysis

Larvae raised to 3dpf were transferred to individual wells of a 96-well plate in a volume of 300 μL E3 media, and maintained overnight for acclimation. The following day (4dpf), larvae were exposed to BAY-293 (10 μM), epinephrine (100 μM), or Crh (5 μg/mL) delivered as 100x stocks (i.e. 3 μL into existing 300 μL E3 in wells), along with the relevant solvent controls (vehicle: ddH_2_O for epinephrine and Crh, or 0.1% dimethyl sulfoxide(DMSO) for BAY-293). In another experiment, larvae were treated with Acth (1 μM) or cortisol (5 μg/mL), with appropriate solvent controls (ddH_2_O for Acth and DMSO for cortisol). Concentrations for all treatments used were selected based on prior studies in teleost models (8, 38–40). Behavioral assessments were carried out between 1200–1500 hours, during the light phase of the 14:10 light:dark cycle, in an isolated room maintained at 28°C. Larvae were subjected to alternating periods (7.5 min duration) of light and dark for 30 min in total as described previously (8). Video of larval movements were captured with the use of the Noldus Danio Vision Observation Chamber, and subsequently analyzed using Ethovision software (Noldus, Leiden, NL). Behavioral experiments were repeated at least three times with independent clutches of embryos collected on different days.

### Acute stressor paradigm

**Figure 1:**
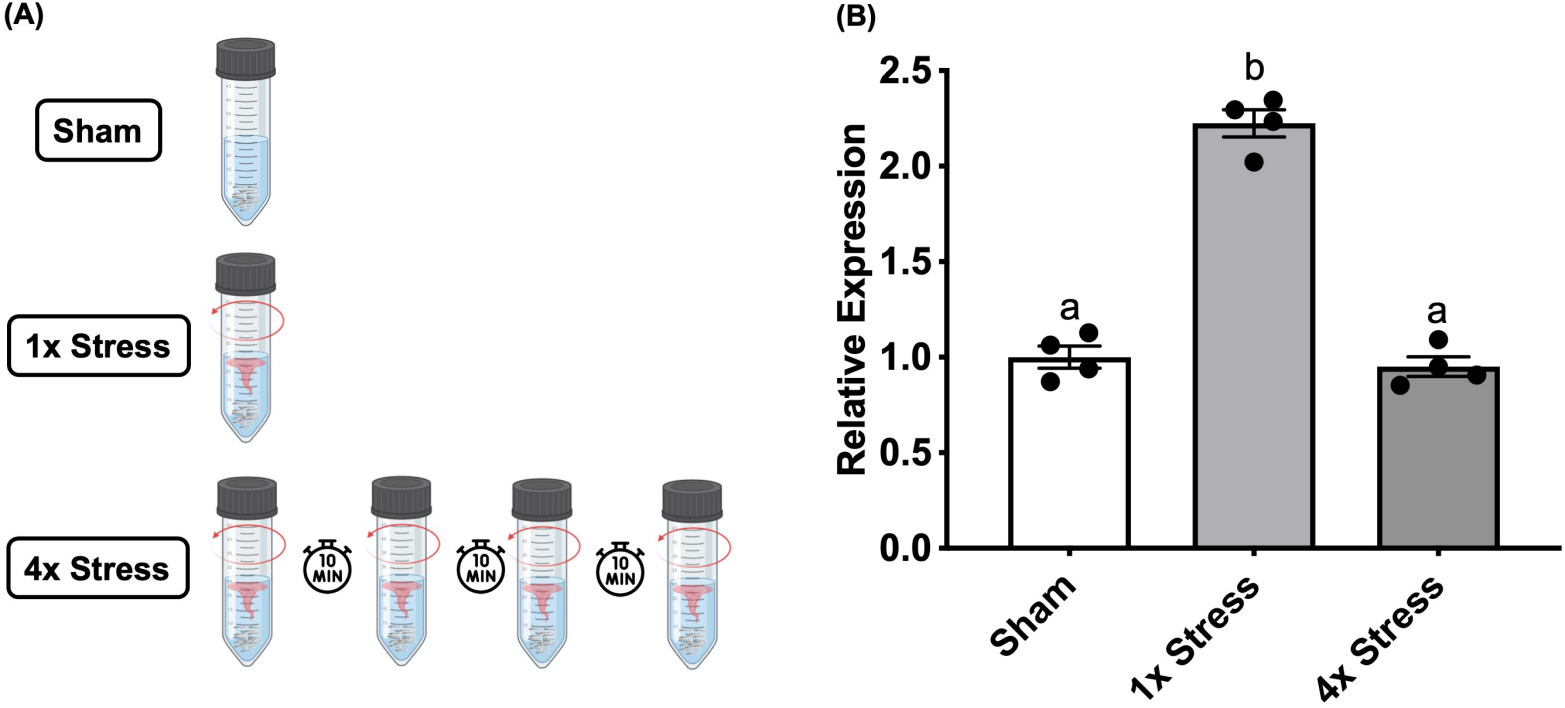
Stressor-induced c-fos expression habituates to repeated homotypic stressors. **(A)** Larvae either received no stressor (sham), a single 1-min stressor (1x), or four consecutive repeated 1-min stressors (4x) spaced apart by 10 min, followed by sampling at 20 min after the final stressor. **(B)** Bars represent mean + SEM (n = 4–10 samples with each sample representing a pool of 10–12 larvae). Data was analyzed by one-way ANOVA followed by Tukey’s multiple comparisons test; p < 0.05.

Larvae were subjected to a swirling stressor as described previously (8). Briefly, larvae raised to 3dpf were transferred to 50 mL centrifuge tubes (VWR, USA), at a density of 25 larvae in 20 mL of E3 media and maintained in the incubator overnight for acclimation. Tubes were assigned to either “Stress” or “Sham” groups, and subdivided based on inhibitor/vehicle treatment. The following day, larvae were pre-treated with BAY-293 (10 μM) or vehicle for 2 h in the same 50 mL tubes. After this treatment period, “Stress” groups were transferred to a heated (28°C) shaker and subjected to a 1-min acute physical swirling stressor (8). Following this stressor, larvae were plated in 96-well plates as described above. “Sham” groups were instead directly transferred to the same 96 well plates without receiving the swirling stressor, and behavioral analysis of all groups proceeded 15 min following plating. Alternatively, for some experiments, larvae were directly sampled from the 50 mL tube at various timepoints following the stressor and rapidly frozen on dry ice for gene analysis (see below). For habituation experiments, larvae were also exposed to repeated swirling stressors to examine the habituation response. Here, a 1-min swirling stressor was followed by a 10-min period of rest, and this was repeated four times, followed by euthanasia and sampling of larvae at 20-min following the final stressor (**Figure 1A**).

### RNA isolation and qPCR

Total RNA was extracted from frozen larval samples using TRIzol reagent (Invitrogen, USA) following manufacturer protocols. Quantity and quality of the extracted RNA was assessed using a Nanodrop 2000 spectrophotometer (Thermo Scientific, MA, USA). 1 μg of RNA was then treated with DNase I (Thermo Scientific) to remove genomic DNA, followed by reverse transcription using a High-Capacity cDNA reverse transcription kit (Applied Biosystems, CA, USA). For quantifying transcript abundance, cDNA template was combined with the appropriate forward and reverse primers (see **Table 1**) and SS Advanced SYBR green (Biorad, Canada), and run in technical duplicates on a QuantStudio 3 Real-Time PCR system (Applied Biosystems). The Quantitative RT-qPCR (qPCR) cycle conditions used were as follows: 94°C for 2 min (polymerase activation), 40 cycles at 95°C for 30 s (denaturation) and 30 s at 60°C (annealing temperatures for all primers used), and lastly 10 min at 72°C (final extension). β-actin was used as a reference gene for normalization, as this transcript was not affected by the treatments. Primer validation and optimization were carried out and annealing temperatures with the highest efficiency was determined prior to RT-qPCR transcript abundance analysis. A melting curve analysis at 65°C to 95°C (5 s) was performed at each qPCR cycle to verify the absence of primer dimers and artifacts for each primer set. The transcript abundance was calculated using the delta-delta CT method (41) as described previously (11).

**Table 1:** List of primer sets used in the present study.

Gene Name	Sequence (5’-3’)	Accession or Reference
c-fos (*fosab*)	F: GTGCAGCACGGCTTCACCGA R: TTGAGCTGCGCCGTTGGAGG	(103)
β-actin (*actb2*)	F: TGTCCCTGTATGCCTCTGGT R: AAGTCCAGACGGAGGATG	(104)
*adra1ab*	F: CAGTTACCCGCTGCAATACC R: CGTTAACTCTGCACACCGAC	XM_021479312.2
*adra1ba*	F: AACTACTGGGTGTTCGGGAG R: CTTCTCGGTCACAATGCTCG	XM_001921978.7
*adra1bb*	F: CCCTTTTACGCGCTCTTCTC R: CCGCAGAGTTATTTCACCGG	NM_001007358.2
*adra1d*	F: AGCATGGGACGGGATTGTTA R: TTTGCTCTTGACGTTGGTGG	XM_691951.7
*adra2a*	F: CTGCAACAGCTCCCTCAATC R: TCCCGTAAACTGCAGGTGAT	NM_207637.3
*adra2b*	F: ATAAAGGTGGTGGCGAGTCA R: GCTGCTTGGCGATCTGATAG	NM_207638.1
*adra2c*	F: ACCAATGTGCCAGCTGAATG R: CCGCCGCTTTTCAGACATAT	NM_207639.1
*adra2da*	F: ATGTTCTTGTGATCGTGGCG R: GCCGAAGTACCAGTATCCCA	NM_194364.2
*adra2db*	F: TCCCACCACTTCTCATGACC R: TGTTTGGCCACCCTGTAGAT	NM_194365.1
*adrb1*	F: GGGTTACTGGTGGTGCCATT R: GCGTGACGCAAAGTACATC	(40)
*adrb2a*	F: GCTTCCAGCGTCTTCAGAAC R: CCGAAGGGAATCACTACCAA	(40)
*adrb2b*	F: CTCGTTCCTACCCATCCACA R: ATGACCAGCGGGATGTAGAA	(40)

### Protein extraction and western blotting

Pools of 25 larvae were sonicated to homogeneity in lysis buffer (50 mM Tris-Cl, 150 mM NaCl, 0.1% Triton X-100) containing both protease and phosphatase inhibitors (Roche, Mississauga, Ontario, Canada). Supernatants were then combined 1:1 with 2x reducing buffer (Laemmli sample buffer with 2β-mercaptoethanol; BioRad) and boiled for 10 min at 95°C. 40 μg of reduced protein lysates were then separated on 10% polyacrylamide SDS-PAGE gels and transferred to 0.45 μm Trans-Blot Pure nitrocellulose membranes (BioRad), followed by PonceauS staining to verify transfer efficiency. Membranes were then blocked for 60 min in blocking buffer (1x Tris-buffered saline and 0.1% Tween 20 (TBS-T) with 5% BSA), followed by overnight incubation at 4°C with primary antibodies against p-ERK (1:2500), total-ERK (1:5000), made up in the same blocking buffer. Following overnight incubation and washes in TBS-T and TBS (3x, 10 min each), membranes were incubated in secondary goat anti-rabbit antibody (1:5000 in TBS- T and 5% skim milk; Gibco, Ontario, Canada) for 2 h at room temperature, after which washes were repeated and membranes imaged using Pierce ECL Western Blotting Substrates (Thermo Fisher Scientific) on a G:BOX Chemi XX6 imaging system (SynGene). Membranes were then stripped using mild stripping buffer (15 g/L glycine, 1 g/L SDS, 1% Tween-2; pH 2.2) prior to re-blocking and probing with anti-β-actin-Cy3 antibody (1:1000) overnight to control for total protein loading within each lane.

### Cryosectioning and phospho-ERK immunohistochemistry

**Figure 2:**
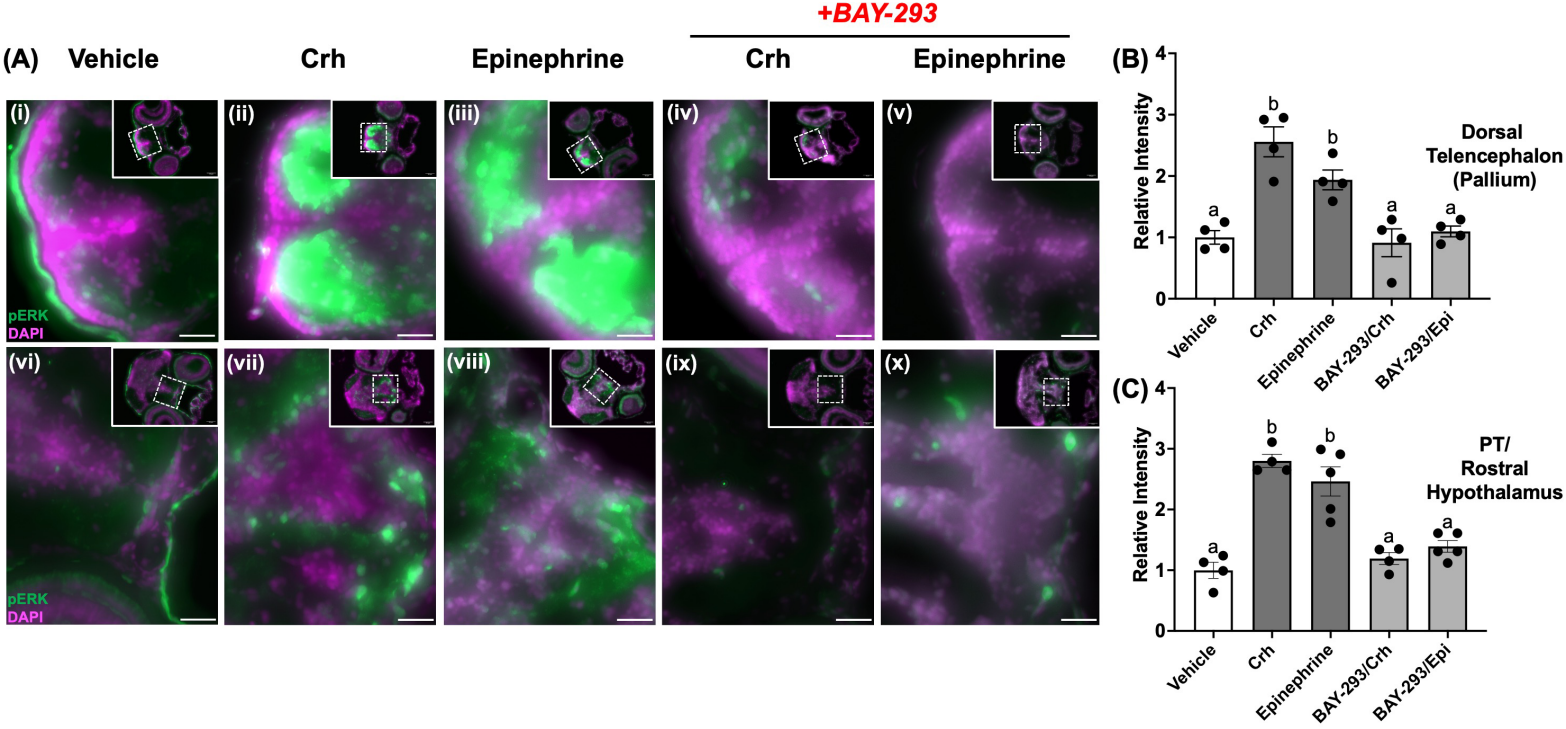
Crh and epinephrine treatments enhance phospho-ERK in discrete larval brain regions. 4dpf WT larvae were treated with either vehicle (dH_2_O, **A, F**), 100 μM epinephrine (upper panel, **B–E**) or 5 μg/mL human Crh **(G–J)** for 10 min. In **(D, E, I, J)**, larvae also received BAY-293 for 2 h prior to acute treatment with epinephrine or Crh. Larvae were then euthanized, fixed, and cryopreserved, prior to sectioning of the brain at 10 μm thickness. Representative photomicrographs show regions of the dorsal telencephalon (pallium) area (upper panels), or slices containing the preoptic area and rostral hypothalamus (lower panels). Insets in each panel show the full slice with the region of interest zoomed in. Merged channels are shown, with green representing phospho-ERK immunoreactive staining, and magenta representing the nuclear co-stain (DAPI). Scale bar: 25 μm. The images were quantified and the relative intensity of phospho-ERK expression in different brain regions are shown on the right. Larval brain sections were analyzed in Fiji for the specific regions of interest, and the integrated density values used to calculate fold change in intensity relative to WT. Bars represent means ± SEM (n = 4-5), with values for individuals shown as data points. Different letters of the alphabet indicate significantly different groups (one-way ANOVA followed by Tukey’s multiple comparisons test; p < 0.05).

4dpf larvae were euthanized and fixed in 4% paraformaldehyde overnight at 4°C and the immunohistochemistry carried out as described previously (14). The following day, larvae were washed in phosphate-buffered saline (PBS) with 0.1% triton-X (PBT), and then incubated in a 30% sucrose solution in PBT for cryopreservation. Larvae were then embedded in OCT (optimum cutting temperature) media (VWR) and stored at -80°C prior to sectioning. Serial transverse sections of the larval brain were then obtained at 10 μm thickness using a Leica CM 3050 S Cryostat (Leica Microsystems, Wetzlar, Germany), and stored on microscopy slides (Superfrost Plus, VWR) at -80 °C until use. Sections were permeabilized with PBS containing 1% Triton (30 min) and blocked with PBS containing 1% BSA and 5% normal goat serum (60 min), and then incubated with primary antibody (1:250 dilution) against phospho-ERK1/2^Thr202/Tyr204^ (Cell Signaling Technologies #4370) overnight at 4°C. Sections were then washed in PBT and exposed to appropriate secondary antibody (Alexa Fluor 488 goat anti-rabbit IgG, Jackson Immunoresearch #111-545-144) at 1:500 dilution for 2 h at room temperature. Sections were then washed with PBT, exposed to DAPI (4′,6-diamidino-2-phenylindole, 0.5 μg/mL) for nuclear staining, and sealed with coverslips until imaging. Fluorescence images were acquired on a Zeiss AxioZoom V1.6 microscope (Zeiss, Germany) using the 2.3x objective. Images containing channels with Alexa Fluor 488 fluorescence (green) or DAPI staining (pink) were analyzed using Fiji software to obtain integrated density values for regions of interest (42). Merged images in **Figure 2** show maximum intensity projection composites for both 488 and DAPI focal planes across an average of 5 brain slices.

### Statistical analysis

Data are presented as mean ± SEM. Statistical testing (one-way or two-way ANOVA, followed by Tukey’s and Holm-Sidak multiple comparisons as a *post-hoc*, respectively) was carried out using Graphpad Prism 10 software, with a *p* value cutoff set to 0.05 for statistical significance. Where appropriate and necessary, raw data was transformed to meet assumptions of normality and homoscedasticity for subsequent statistical testing. Untransformed data is presented in figures.

## Results

### Stressor-induced light-phase hyperactivity requires active Ras-MAPK

**Figure 3:**
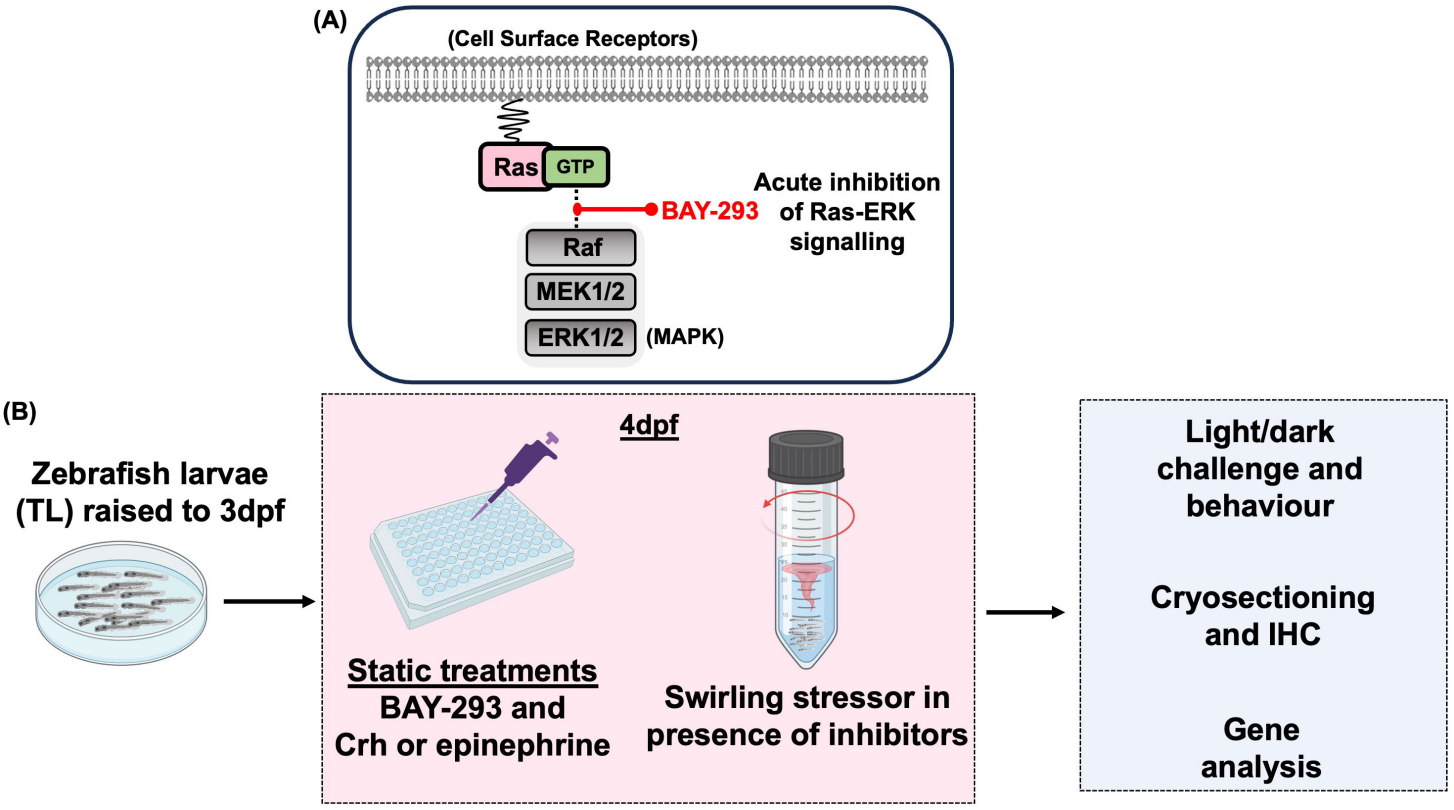
Experimental overview schematic. **(A)** BAY-293 is a specific inhibitor of Ras-MAPK signaling and used in this study to pharmacologically inhibit this pathway and study acute stress-related behavioral and gene responses. **(B)** For all experiments, zebrafish larvae were raised to 3dpf in petri dishes prior to being transferred to plastic plates or falcon tubes for 4dpf experiments, depending on the specific protocol (see Methods). For static treatments, larvae were exposed to BAY-293 and stress hormones and either sampled for gene analysis or immunohistochemistry or challenged with the light-dark paradigm to investigate stress-related behaviour. Alternatively, 4dpf larvae in falcon tubes underwent a 1-min swirling stressor with or without pre-treatments, followed by light-dark behavioral analysis and/or sampling for gene expression.

**Figure 4:**
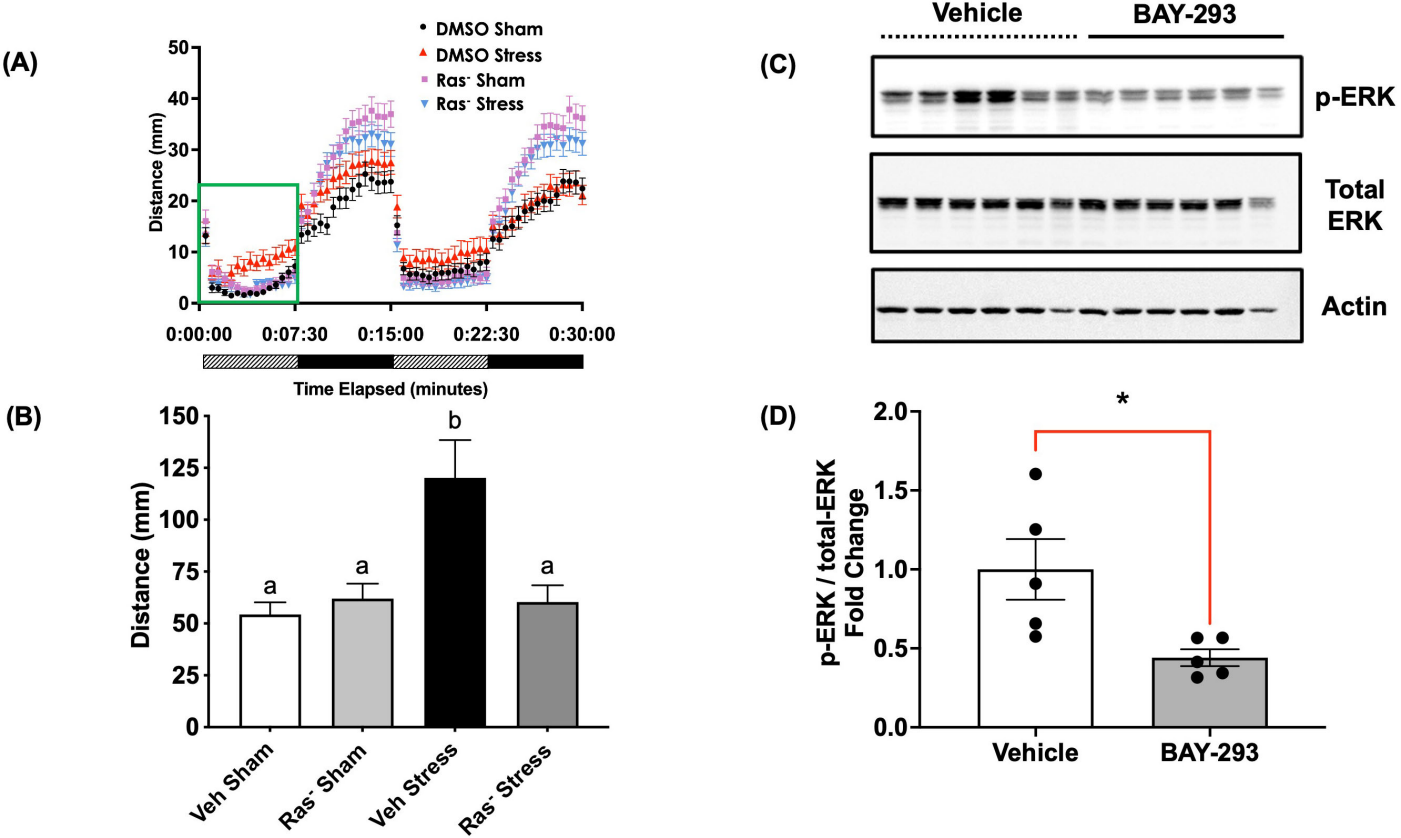
Blockade of Ras signaling attenuates the acute stressor-induced behavioral response. **(A)** 4dpf WT larvae were pre-treated in 50 mL centrifuge tubes for two hours with either vehicle (0.01% DMSO final concentration) or 10 μM BAY-293 (“Ras^-^” groups), prior to exposure to a 1-min physical swirling stressor (“Stress” groups). “Sham” groups were maintained in the same centrifuge tubes but did not receive the swirling stressor. All larvae were then transferred to 96-well plates and exposed to alternating periods of light and dark (7.5 min duration x 2 cycles). Locomotion over all four phases is presented in **(A)**, and **(B)** shows the mean summated values of distance moved in the first light phase for all treatment groups. Error bars represent mean + SEM and bars with different letters are significantly different (one-way ANOVA followed by Tukey’s multiple comparisons test; p < 0.05; n = 113-129). **(C)** pools of 4dpf WT larvae (25 larvae) were treated with vehicle or BAY-293 for 2 h, followed by protein extraction and probed for phospho- or total-ERK1/2 immunoreactive proteins. BAY-293 treatment specifically reduced phospho-ERK signal while total-ERK was unaffected. β-actin was used as a loading control. **(D)** Band intensity values for phospho-ERK and total-ERK were first normalized to β-actin and then calculated as a fold change. Lanes 6 (Vehicle) and 12 (BAY-293) were excluded from the analysis due to the change in β-actin levels. Error bars represent mean ± SEM (Student's t-test; *, p < 0.05; n = 5).

4dpf zebrafish larvae underwent a 1-min swirling stressor protocol followed by assessment of the stress-related behavioral response as described previously (43) (**Figure 3**). Briefly, the light-phase “freezing” response in 4dpf larvae is changed to hyper locomotory activity after an acute stress and previously validated to represent stress-related behaviour (8, 14). Here, the control larvae subjected to an acute stressor demonstrated elevated light-phase activity (**Figure 4A**). However, pre-treatment with the Ras-MAPK inhibitor BAY-293 (10 μM) inhibited this hyperactivity response, restoring locomotory distance to levels not different from the unstressed controls (**Figure 4A**). To verify that BAY-293 indeed acts on the Ras pathway in zebrafish larvae, we confirmed that treatment of 4dpf larvae with this compound specifically reduces phospho-ERK^(Thr202/Tyr204)^ immunoreactivity in protein lysates collected from whole larvae by ~55% (**Figures 4B, C**).

### Crh and epinephrine treatments rapidly induce phospho-ERK in larval brain regions

We next evaluated whether stress hormones could alter ERK phosphorylation status in key brain regions that may underlie the observed behavioral changes observed. To assess this, we probed the induction of phospho-ERK in the 4dpf larval brain, following 10-min treatments with Crh or epinephrine, the canonical initiators of the endocrine and sympathetic stress axes, respectively. Treatment with either hormone strongly induced phospho-ERK in the dorsal pallium region (**Figure 2Ai-iii**), as well as in several nuclei in the preoptic/rostral hypothalamic area (**Figures 2Av-vii**). In the pallium, treatment with Crh and epinephrine elevated phospho-ERK levels relative to vehicle by 2.5 fold and 1.9 fold, respectively (**Figure 2B**). Similarly, treatment with Crh and epinephrine elevated phospho-ERK levels relative to vehicle by 2.8 fold and 2.4 fold, respectively, in the posterior tuberculum/rostral hypothalamus areas (**Figure 2C**). Importantly, this induction of phospho-ERK in both regions could be prevented by pre-incubation with BAY-293 (2 h) (**Figures 2Aiv, v, ix, x** and **Figures 2B, C**), confirming that Crh and epinephrine activate phospho-ERK through the Ras-dependent pathway in larval brain regions.

### Crh and epinephrine drive the acute hyperactivity response through Ras-MAPK signaling

**Figure 5:**
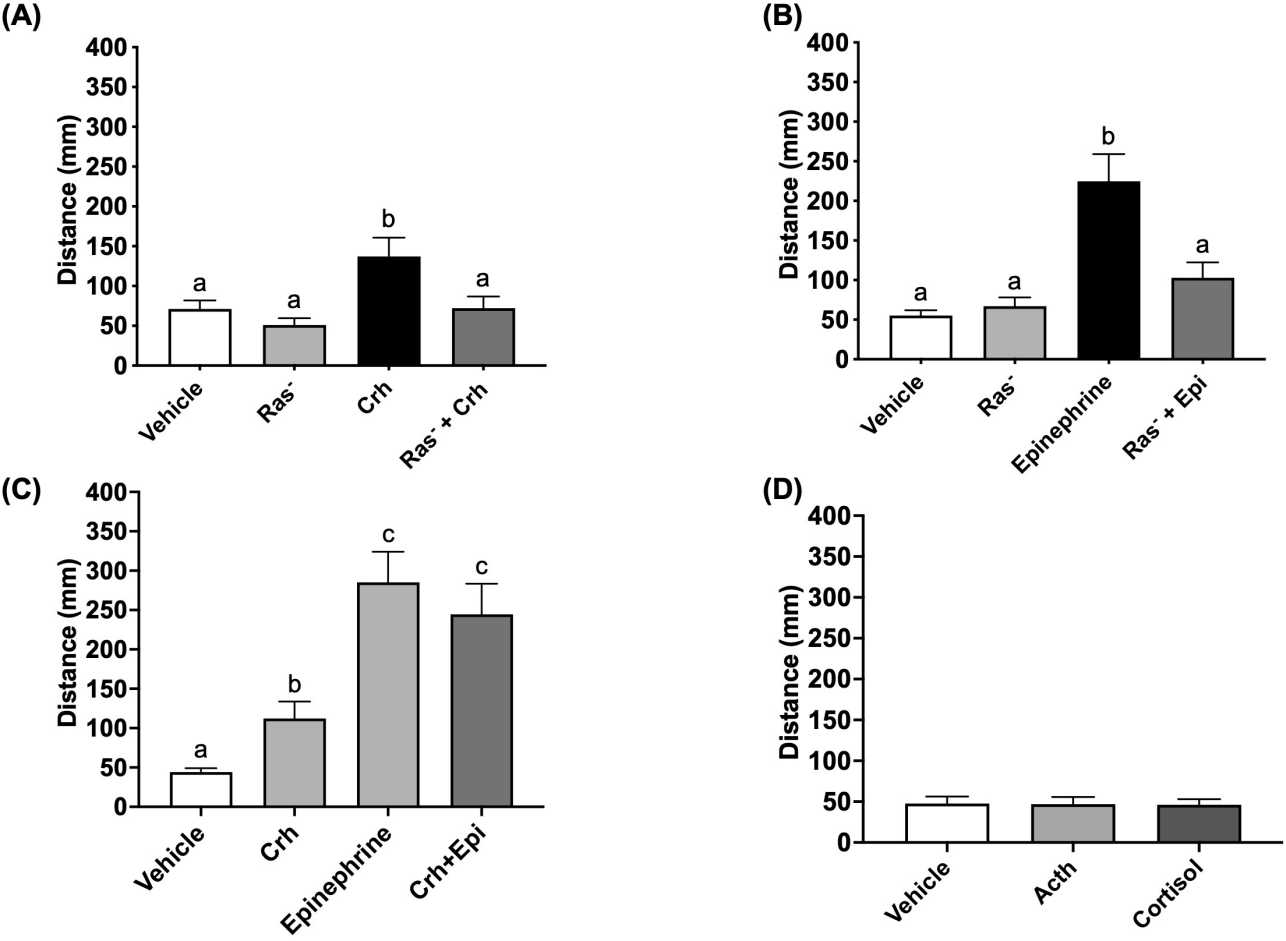
Crh and epinephrine utilize the Ras pathway to drive acute stress-related hyperactivity. 4dpf WT larvae were pre-treated with vehicle (0.01% DMSO final concentration) or 10 μM BAY-293 (“Ras^-^” groups) in 96 well plates for 2 h, prior to being acutely treated with either epinephrine (**A**, 100 μM) or human Crh (**B**, 5 μg/mL) for 15 min. Larvae then underwent light-dark challenge and behavioral analysis (7.5 min duration of light/dark x 2 cycles). In **(C)**, larvae were treated with Crh (5 μg/mL), epinephrine (100 μM), or a combination of the two, prior to the same light-dark challenge. Finally, in **(D)**, larvae were treated with adrenocorticotropic hormone (ACTH, 1 μM) or hydrocortisone (cortisol, 5 μg/mL) for 15 min prior to the light-dark challenge. Finally, Graphs in A-D show mean summated values of distance moved in the first light phase for all treatment groups. Error bars represent mean + SEM with different letters of the alphabet indicating significantly different groups (one-way ANOVA followed by Tukey’s multiple comparisons test; p < 0.05; n = 70–108 for **(A)**; n = 56–60 for **(B)**; n = 50–51 for **(D)**; Kruskal-Wallis test for **(C)**, n = 78-95).

Given that acute stressor induced the behavioral hyperactivity response in a Ras-Mapk-dependent manner, we sought to determine whether canonical stress hormones involved in the acute stress response would utilize this key signal transduction pathway. As demonstrated before (8), treatment with Crh or epinephrine reliably stimulated light-phase hyperactivity in 4dpf larvae (**Figures 5A, B**), to levels 2.2-fold and 4-fold higher than vehicle controls, respectively. However, in the presence of BAY-293, this behavioral response was abrogated in both cases, demonstrating the engagement of the Ras-Mapk pathway by epinephrine and Crh as a necessity for the acute stressor-induced locomotory response. Since Crh- and epinephrine-driven hyperactivity was similarly dependent on active Ras-Mapk signaling, we next tested whether the pathway was saturated or could further drive locomotor activity in the presence of both hormones together. Here, larvae received either Crh, epinephrine, or a combination of Crh and epinephrine and underwent light-dark challenge and behavioral analysis 15 min post-treatment. While Crh and epinephrine individually elevated light-phase locomotion, the epinephrine response was greater than 2-fold compared to the Crh response (**Figure 5C**). However, the combination group did not further elevate the hyperactivity above that seen with the epinephrine treatment (**Figure 5C**).

### The acute hyperactivity response is independent of Acth or cortisol actions

Since Crh was shown to induce light-phase hyperactivity, it was necessary to determine how and whether the canonical downstream elements of the HPI axis (i.e., pituitary Acth release and interrenal cortisol secretion) were involved in facilitating this acute behavioral response. Larvae were treated with Acth (1 μM) or cortisol (5 μg/mL) for 15 min and the locomotory response to light tested as described above for Crh stimulation. However, neither of these hormones were able to induce acute hyperactivity, suggesting that the effects of Crh are specific and independent of the downstream action of HPI axis stimulation (**Figure 5D**, **Supplementary Figure S1**).

### Epinephrine-induced locomotory response is modulated by Crhr1

**Figure 6:**
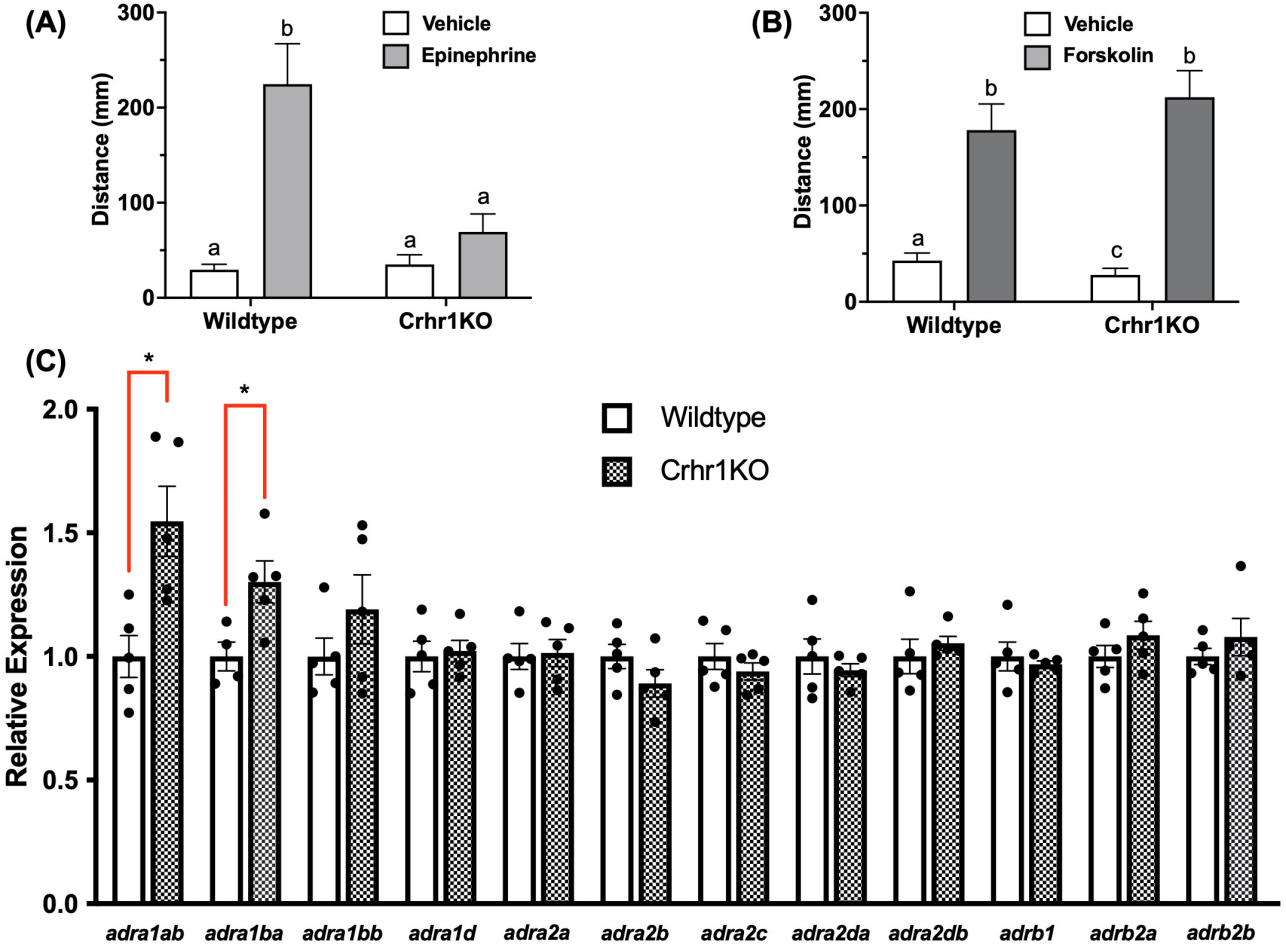
Crhr1 modulates epinephrine response. In **(A, B)**, 4dpf WT or crhr1-/-larvae were exposed to epinephrine (100 mM) or vehicle (dH2O) **(A)**, or forskolin (10 mM) or vehicle (0.1% DMSO) (B) for 15 min, prior to light-dark challenge and behavioral analysis (7.5 min duration of light/dark x 2 cycles). Bar graphs in A and B show mean summated values of distance moved in the first light phase for all treatment groups. Error bars represent mean + SEM with different letters of the alphabet indicating significantly different groups (two-way ANOVA followed by Holm-Sidak’s multiple comparisons test; p < 0.05; n = 66-72). In **(C)**, untreated 4dpf WT or crhr1-/-larvae were sampled for analysis of adrenoceptor transcript abundance. An asterisk indicates a statistically significant difference between genotypes (Student’s t test; *: p<0.05).

To explore potential interactions between the sympathetic and HPI stress signaling, we subjected both WT and *crhr1^-/-^* larvae to epinephrine treatment prior to the light/dark challenge and behavioral analysis. While 15-min epinephrine treatment reliably induced light-phase hyperactivity in WT larvae, it failed to increase locomotory activity in the *crhr1^-/-^* larvae relative to vehicle controls (Two-way ANOVA, p = 0.86, **Figure 6A**). To test whether this defect in locomotion was possibly due to changes in intracellular signaling, we treated both WT and *crhr1^-/-^* larvae with forskolin, an activator of adenylate cyclase, a major signaling pathway utilized by adrenergic receptors (44). Following 15-min forskolin treatment, both WT and *crhr1^-/-^* larvae displayed comparable levels of light-phase hyperactivity (Two-way ANOVA, p = 0.83, **Figure 6B**). Lastly, to test whether the observed differences in responses to epinephrine in larvae lacking Crhr1 may be at the receptor level, we compared expression of a suite of alpha (α) and beta (β) adrenoceptor genes between 4dpf WT and *crhr1^-/-^* larvae. Here, we found that in the absence of Crhr1, larvae exhibited higher transcript abundance of two α_1_-adrenoceptor subtypes, *adra1ab* and *adra1ba*, as compared to WT counterparts (Student’s t-test, p < 0.05, **Figure 6C**). No changes in transcript abundances were observed for any other α_2_,β_1_, or β_2_ adrenoceptor subtypes (**Figure 6C**).

### c-fos is a downstream target of the ERK pathway during stress

**Figure 7:**
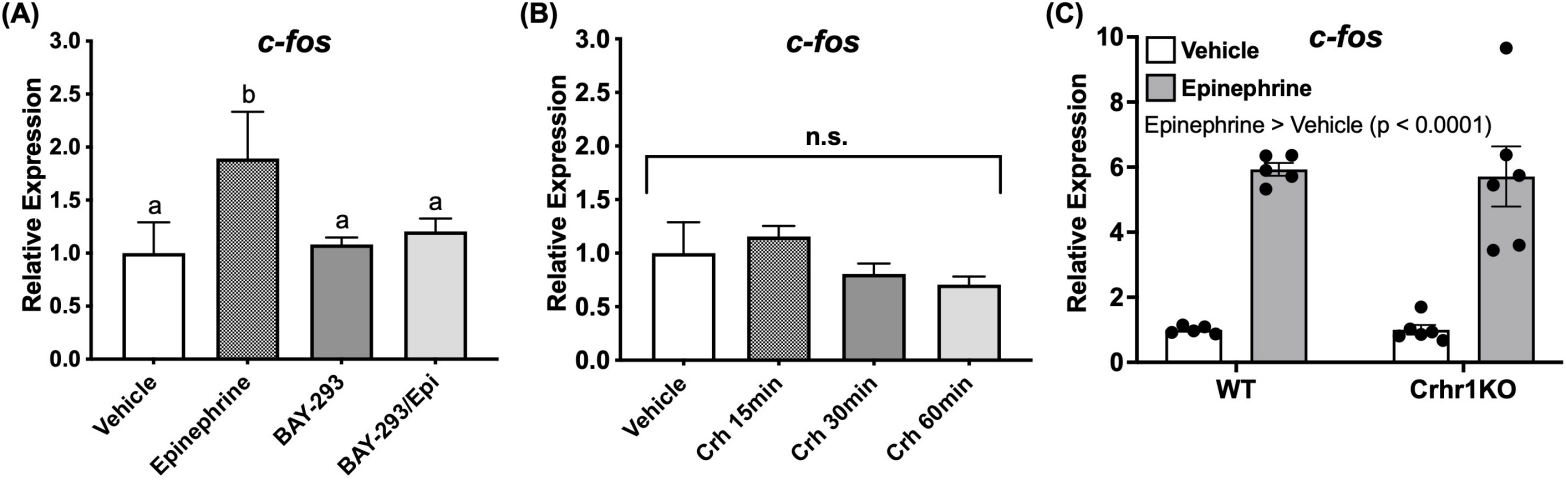
Epinephrine, but not Crh affects c-fos transcript abundance. 4dpf WT larvae were treated in 6 well plates with epinephrine **(A)** or Crh **(B)** and sampled at multiple timepoints for analysis of c-fos transcript abundance with or without BAY-293 (10 mM) pre-treatment (2 h). In panel (C), 4dpf WT or crhr1-/-larvae were treated in 6 well plates with epinephrine and sampled at 1 h for analysis of c-fos transcript abundance. Bars represent mean + SEM (n = 4–10 samples, with each sample representing a pool of 10–12 larvae). For (A), different letters of the alphabet indicate significantly different groups (one-way ANOVA followed by Tukey’s multiple comparisons test; p < 0.05). In (C), groups were compared using two-way ANOVA followed by Holm-Sidak’s multiple comparisons test; p < 0.05.

Having shown that Crh and epinephrine drive changes in behavior and induce ERK phosphorylation in key brain regions, identifying downstream targets of this signaling pathway in the brain was essential. Among the many substrates of ERK, one well-established neuronal marker known to act downstream is the immediate early gene *c-fos*, which is rapidly induced during sustained neuronal activity (30, 31). We observed that larvae treated with epinephrine showed elevated transcript abundance of this gene in whole-body extracts in a Ras dependent manner, since BAY-293 pre-treatment prevented induction of *c-fos* (**Figure 7A**). However, Crh treatments tested at multiple timepoints failed to show a similar upregulation of this gene (**Figure 7B**). Furthermore, despite the lack of epinephrine effect on locomotory behavior in *crhr1^-/-^* larvae (**Figure 6A**), epinephrine treatment was still able to induce *c-fos* expression in larvae lacking Crhr1 compared to the WT (Two-way ANOVA, **Figure 7C**).

### Acute stress stimulation of c-fos is independent of Crhr1

**Figure 8:**
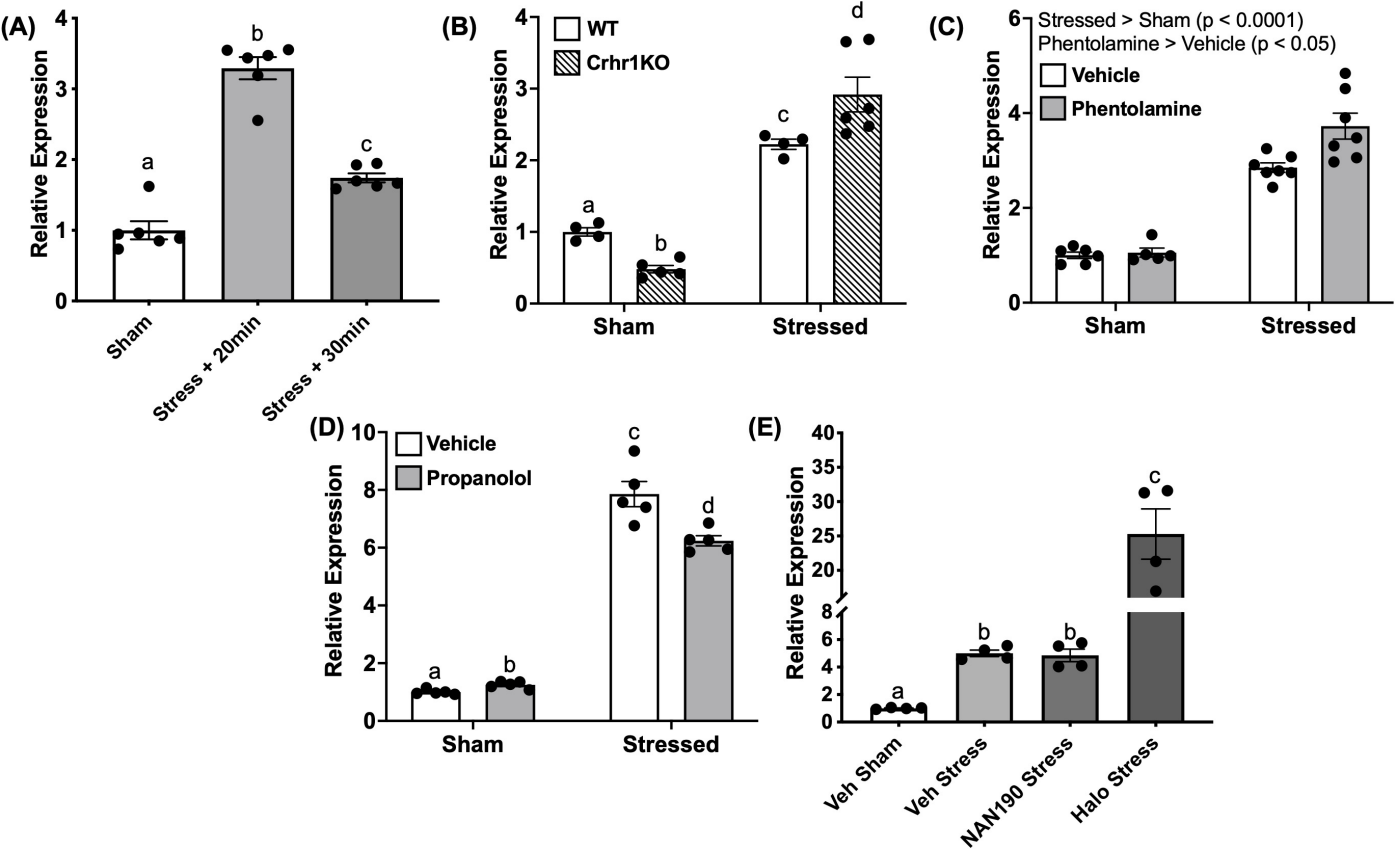
Acute stressor modulates c-fos transcript abundance. In **(A)**, 4dpf WT larvae underwent the 1-min stressor protocol in 50 mL falcon tubes and were then sampled at 20 and 30 min post-stressor. This was repeated using larvae lacking functional Crh receptor 1 (crhr1-/-) in **(B)** In **(C–E)**, larvae were pre-treated for one hour with receptor antagonists for a-adrenoceptor (phentolamine, 100 mM), b-adrenoceptor (propranolol, 100 mM), 5HT1A receptor (NAN-190, 100 nM), or dopamine D2 receptor (haloperidol, 20 mM) prior to the 1-min swirling stressor and then sampled at 20 min poststressor. In all panels, bars represent mean + SEM (n = 4–10 samples with each sample representing a pool of 10–12 larvae). Different letters of the alphabet indicate significantly different groups (A, E: one-way ANOVA followed by Tukey’s multiple comparisons test; p < 0.05). For (B–D), data were analyzed via two-way ANOVA followed by Holm-Sidak’s multiple comparisons test. Main effects are reported within the figure panel, and different letters of the alphabet indicate significant differences when interactions are present.

We next tested whether exposure to an acute stressor could induce *c-fos* expression. Subjecting larvae to the swirling stressor transiently increased *c-fos* expression at 20 min (3.2 fold *vs*. sham), which was reduced at 30 min post-stress, but still significantly higher than the pre-stress values (1.7 fold *vs.* sham) (**Figure 8A**). Thus, the 20 min timepoint was chosen for subsequent experiments characterizing *c-fos* induction. To assess whether Crh played a role in the acute stressor-induced *c-fos* expression, we exposed *crhr1^-/-^* larvae to the acute stressor. Stressor-induced *c-fos* expression seen in the WT persisted in the *crhr1^-/-^* larvae compared to sham controls (Two-way ANOVA, p < 0.0001). The *crhr1^-/-^* larvae also showed a greater *c-fos* transcript abundance post-stressor compared to the WT group (Two-way ANOVA, p < 0.0001, **Figure 8B**). Basal *c-fos* was also significantly lower in the *crhr1^-/-^* compared to the WT (Two-way ANOVA, p < 0.0001, **Figure 8B**).

To test for SNS involvement in the stressor-induced c-fos response, we treated WT larvae with inhibitors of adrenergic receptors, prior to the acute stressor exposure. Pre-treatment with phentolamine (α-adrenoceptor antagonist) elevated (1.3 fold) *c-fos* expression compared to the vehicle (Two-way ANOVA, main effect, p < 0.05, **Figure 8C**). On the other hand, propranolol (β-adrenoceptor antagonist) treatment reduced (1.3 fold) the acute stressor-induced *c-fos* expression compared to stressor vehicle controls (Two-way ANOVA, p < 0.01, **Figure 8D**). The monoamine neurotransmitter receptors, including 5HT_1A_ receptors (45, 46) and D_2_ receptors (47, 48), are known to play a role in the acute stress-related behavioral responses. Pre-treatment with NAN-190 (5-HT_1A_ receptor antagonist) did not affect the stressor-induced *c-fos* abundance, while haloperidol (antagonist of dopamine D_2_-like receptors) resulted in a robust (5 fold) elevation of *c-fos* transcript abundance post-stressor compared to vehicle controls (One-way ANOVA, p < 0.0001, **Figure 8E**).

### Role of c-fos in the acute stressor habituation response

Stressor-dependent *c-fos* responses are known to diminish with repeated stressor due to neuronal habituation in mammalian models (34–36). To assess whether *c-fos* has a similar role in habituation in our larval zebrafish stress paradigm, we applied a multiple/repeated stressor to WT larvae, where larvae received the 1-min swirling stressor followed by a 10-min rest period, with this process then repeated four times (**Figure 1A**). Larvae were sampled at 20-min following the final stressor. We recently observed that this repeated stressor paradigm results in a suppression of the stressor-induced locomotory hyperactivity response, with larvae that received 4 stressors displaying light-phase locomotion similar to unstressed/sham controls (J.J. Rajeswari, G.N. Gilbert and M.M. Vijayan, *in prep*). Here, we assessed the *c-fos* response at 20-min following either a single stressor (1x) or four repeated stressors (4x), and found that *c-fos* levels mirror the behavioral response, in that it is no longer elevated following multiple stressors (One-way ANOVA “4x stress” vs sham, p = 0.83, **Figure 1B**).

## Discussion

In teleosts, as in mammals, it has been well-established that acute rapid stress responses are driven predominantly by activation of the SNS, while the slower acting and sustained stress responses are mediated by the canonical HPA/HPI axis (2, 4, 10, 49). While the longer-term transcriptional effects of cortisol are well-understood, the signaling changes and molecular mechanisms underlying the rapid changes during the initial neuropeptide phases of the HPI axis activity have received less attention. Using the zebrafish model, our group previously reported the behavioral light-phase hyperactivity response to represent an acute stress-related behaviour (8, 50). Here, we took advantage of the ubiquitous Crhr1 knockout model (8) and whole-organism Ras-Mapk signaling inhibition to gain insight into the intracellular signaling pathway utilized by Crh-Crhr1 system in regulating the rapid stress-related locomotor activity in zebrafish larvae. Our results reveal that Crhr1 usage of the Ras-Mapk pathway as a central mechanism regulating the acute stress-induced hyperactivity in zebrafish larvae. Furthermore, Crhr1 has a complementary but essential role in modulating the epinephrine-mediated fight-or-flight response, but not the c-fos response.

The Ras GTPase serves as a key linker of the canonical three-tiered MAPK modules to cell-surface receptors from several families, placing it in a position of prominence in initiating cellular responses to diverse extracellular stimuli, including most neurotransmitters and hormones (15, 51). In this study, we used an inhibitor of the Ras-MAPK cascade, BAY-293, to understand how this crucial signaling arm affects stress-related behavioral responses (52) in zebrafish larvae. Treatment with BAY-293 reduced levels of phospho-ERK in larval protein extracts, consistent with the targeted specificity of this inhibitor (52) and demonstrated previously using *in vitro* models (39) from closely-related cyprinid fish species (*Carassius auratus*). Here we show that the stressor-induced locomotor activity was completely abolished by BAY-293, indicating a key role for the Ras-MAPK cascade in the initiation of the acute stress-related hyperactivity in zebrafish larvae. We next investigated whether Ras-MAPK signaling in central brain regions may be tied to the observed stress-related behavioral effects. Interestingly, multiple regions showed elevated phospho-ERK activation in response to acute treatments with Crh or epinephrine (**Figure 2**). In particular, cell populations in the telencephalic pallium region were robustly activated by both hormones in a Ras-dependent manner (**Figures 2i-v**). This region of the telencephalon has a critical role in a broad range of learning and behavior-related processes, including in the context of stress (53, 54), and contains regions homologous to the mammalian hippocampus and amygdala (55, 56). The finding that epinephrine and Crh signaling rapidly activates cell populations in these regions is likely indicative of stress-related learning and control of downstream behavioral outputs. As well, we found that Crh and epinephrine also induced phospho-ERK activation in brain slices containing the preoptic and rostral hypothalamic regions (**Figures 2vi-x**). Nuclei in these areas coordinate a diverse range of homeostatic regulatory functions (57–59), including acute stress-related survival behaviors in teleosts, consistent with the present observations (60, 61). This suggests that the early fight-or-flight responses are modulated by both epinephrine and Crh and may impinge upon these circuits as part of modulating the homeostatic responses to stress, including driving defensive behaviors. Follow-up studies with selective markers of neuronal populations in both hypothalamic and telencephalic regions will be necessary to shed further light on the specific neural processes involved. Furthermore, phospho-ERK induction indirectly reflects only one discrete timepoint of neuronal activity; thus, to build on this initial screening experiment, future work will need to incorporate live readouts such as Ca^2+^ activity for a clearer picture of neuronal activation dynamics in response to Crh and epinephrine (62). Beyond the Ras-dependent behavioral changes observed, it is likely that similar pathways are engaged to support the energy substrate allocation for rapid locomotion, especially in the case of stimulation with epinephrine. While not a primary focus of this study, acute Ras-ERK signaling is also involved in peripheral tissue action, including skeletal muscle and vascular smooth muscle, which may also contribute to the observed behavioral responses (63, 64).

In mammals, the Ras-MAPK module is a well-known transducer of adrenergic receptor signaling (65), and Crhr1 signaling also leads to activation of ERK (37, 66, 67). A recent study suggested that fish (*Larimichthys crocea*) Crhr1 heterologously expressed in HEK293 cells are also capable of activating downstream ERK phosphorylation when stimulated with LcCrh (68). Together with our results demonstrating this for the first time in an *in vivo* model, this supports the notion that the intracellular signaling outputs of Crhr1 are conserved across vertebrate taxa. Importantly, we have demonstrated the functional relevance of this signaling endpoint *in vivo* using the stress behavioral hyperactivity readout, suggesting a direct role for this receptor activation in the zebrafish flight response to stress, which may complement the actions of epinephrine. In mammals, Crh is known to interact with the sympathetic system either at a network level upstream of the locus coeruleus (69–72), or through direct downstream interactions with adrenoceptors (73). These interactions are thought to fine-tune stress-induced responses and allow for behavioral flexibility to deal with various challenges (69, 71). Evidence suggests that the Crh-Crhr1 system can influence behavioral responses in both mammals (74, 75) and teleosts such as chinook salmon *(O. tshawytscha*) (76) and rainbow trout (*O. mykiss)* (77).

Notably, the effects observed on behavioral responses here were independent of downstream HPI elements including Acth, cortisol, and changes in corticosteroid receptor expression. While chronic cortisol (treatment for several hours) elicits light-phase hyperactivity in the long term, likely through transcriptional events (43), acute treatments with either cortisol or Acth did not alter locomotory behavior in the present study. This is consistent with growing evidence of the rapid neuro-modulatory roles of Crh in behavioral and stress/anxiety-related responses in mammals, which are initiated either prior to or in parallel with downstream HPI endocrine activity (74, 75, 78). In particular, it has been proposed that behaviors following exposure to an acute stress may be driven by branching collaterals of PVN (paraventricular nucleus in mammals) Crh neurons to both intra- and extra-hypothalamic circuits (78). These neuronal projections, ranging from proximal hypothalamic sites all the way to distant nuclei of the brainstem, underlie the ability of PVN^CRH^ neurons to orchestrate behavioral outputs on an accelerated timescale relative to downstream endocrine actions, adding a complex repertoire of actions to these neuroendocrine cells beyond their classical functions in stimulating pituitary Acth release (75). Our results are consistent with this notion of acute Crh actions as a highly conserved rapid response pre-empting the downstream cortisol biosynthesis for stress adaptation.

While the stress-induced behavioral response was mimicked by treatment of larvae with either Crh or epinephrine (**Figure 5**), the magnitude of response was >2-fold with epinephrine compared to Crh, suggesting a greater SNS stimulation of the behavioral response. However, it is interesting to note that this epinephrine-mediated locomotory response was completely abolished in larvae lacking Crhr1 (**Figure 6A**) pointing to a central role for the Crh-Crhr1 system in modulating the SNS activation of the locomotory response. This may be associated with adrenoceptor regulation by Crhr1 as forskolin was able to bypass the inhibitory effect of epinephrine on the locomotory response. Indeed, there were genotype differences in the transcript abundance of adrenoceptor α_1A_ and α_1B_ paralogs between WT and *crhr1^-/-^* larvae (**Figure 6C**). Both *adra1ab* and *adra1ba* are known to be predominantly expressed in zebrafish brain, as compared to other tissues including the heart, liver, muscle, kidney and gill (79). While specific functional data regarding these subtypes are lacking in fish, rodent studies have revealed their roles in driving various aspects of arousal, and sensorimotor control, in the context of the stress response (80–82). As well, these receptors exhibit both unique and overlapping expression patterns in areas such as the hippocampus, thalamus, and cortex (80, 83). Overall, α_1_ subtypes tend to induce synaptic depression in the brain (84). In particular, the α_1A_ subtype is known to mediate noradrenergic modulation of hippocampal excitability, specifically increasing inhibitory tone through GABAergic and somatostatinergic systems, which is relevant in the context of epilepsy (85). α_1B_ adrenoceptors also demonstrate a wide distribution throughout the rat brain and are posited to play key roles in stress, neuroendocrine, and motor functions (80, 86). Taken in context with the present results, the altered transcript abundance of these receptor isoforms in *crhr1^-/-^* larvae may underlie the lack of locomotory response to epinephrine stimulation. This suggests a role for these receptors in the locomotory response to epinephrine stimulation in zebrafish larvae. Future studies would benefit from examining the expression of these proteins from a functional standpoint; however, the lack of antibodies for these receptors in zebrafish is a major drawback.

Given the Ras-dependent modulation of behavioral endpoints triggered by Crh and epinephrine actions, together with the ERK phosphorylation in specific brain regions, we sought to identify a neuronal marker as a downstream target of Ras signaling in this context that may represent stress-related learning. We chose to assess *c-fos* as a potential target, as it is a well-characterized immediate early gene (IEG) that is induced following sustained neuronal activity and depolarization (31). The protein product Fos is a component of the AP-1 complex which acts as a transcription factor regulating expression of a diverse set of genes (32, 33). In addition, both gene and protein products of *c-fos* have been commonly utilized in stress studies as a marker of stressor-induced neuronal activation in response to diverse types of stressors, where *c-fos* expression may be indicative of learning and memory in relation to specific stressor contexts (87–89). Indeed, we also observed induction of the *c-fos* gene following exposure to an acute stressor, further validating its use as a marker for stressor-induced responses. We show for the first time the differential involvement of the stress hormones epinephrine and Crh in engaging the *c-fos* response; that is primarily mediated by epinephrine and not by Crh stimulation, indicating a divergence in intracellular signaling following the common activation of the Ras-MAPK pathway by these two hormones (**Figure 7**). Also, despite epinephrine failing to stimulate hyperactivity in *crhr1^-/-^* larvae, the epinephrine-induced *c-fos* response persisted (**Figure 7C**), suggesting that both these effects mediated by epinephrine may be independent, potentially due to a temporal uncoupling of signaling pathways. It remains to be determined if this uncoupling is due to the regulation of distinct sets of receptor subtypes by Crhr1, given that the epinephrine-induced hyperactivity was abolished in the Crhr1 knockout model. In WT larvae, blockade of β-adrenoceptors, but not α-adrenoceptors, could inhibit this stress-dependent *c-fos* expression. Given the small (yet significant) magnitude of this effect, other receptors besides β-AR, possibly acting in specific combinations, may likely be involved in driving the *c-fos* response, but this remains to be determined.

Despite being a common signaling target, it is known that ERK dynamics can influence cellular responses in distinct fashions depending on the specific agonist/stimulus (15, 90) and targeted follow-up work may help identify specific substrates of phosphorylated ERK1/2 in the Crhr1-activated context in zebrafish larvae. Interestingly, some work in mammals has shown primarily cytoplasmic/perinuclear localization of p-ERK in hippocampal cells following Crh administration or fear conditioning, which may explain the lack of effect on *c-fos* induction by zebrafish Crhr1 (37, 91). The lack of effect of Crh on *c-fos* also suggests the involvement of distinct Mapk/Erk substrates following Crhr1 activation that was previously unexplored in fish. Underlying this variance may be unique receptor-proximal mechanisms utilized by Crhr1 and the adrenergic receptors leading to intracellular signaling, such as the involvement of different G protein subunits, as well as other transducers such as β-arrestins and downstream protein kinases. Briefly, both of these hormone-receptor systems are capable of engaging multiple Gα subunits and/or arrestins, and consequently distinct downstream pathways depending on the tissue context (92–95). Thus, despite a general convergence onto ERK signaling, downstream consequences of this activation may be distinct through differential targeting of unique ERK targets in both the cytosol and nucleus (96). Temporal encoding is also a feature of the ERK pathway, with differences in *c-fos* and other IEG induction arising from pulsatile *vs.* sustained modes of stimulation (97). Overall, information on many of these nuanced signaling mechanisms are generally lacking for both these receptor systems in fish species and will require targeted follow-up studies to clarify.

In addition to SNS activation, other neurotransmitter systems appear to be capable of modifying the stressor-induced *c-fos* response in mammalian systems (98, 99). While antagonism of 5HT_1A_-type receptors (NAN-190) had no impact in our study, pre-treatment with haloperidol (antagonist of dopamine D_2_-like receptors) resulted in a pronounced elevation of *c-fos*, indicative of a strong negative regulation of dopamine over this stressor-induced response (**Figure 8E**). In the mammalian literature, dopaminergic pathways, especially those mediated by D_2_R, are known to play several key roles in inhibitory processes, including response inhibition in both cognitive and motor contexts; this is exemplified by a lack of such control in pathologies related to imbalances in the dopaminergic system (100, 101). Given that this role is generally conserved across vertebrate evolution (102), it is tempting to speculate that the release of D_2_R inhibition through receptor antagonism drives the resulting increase in stress-induced neuronal activation as measured through levels of *c-fos*. However, as already mentioned, future work utilizing specific neuronal markers will be important in determining spatial localization of the *c-fos* response as well as in elucidating the identity of the neuronal populations being activated in response to our acute stressor paradigm. We also observed in the present study that the stressor-induced *c-fos* response is diminished following repeated swirling stressors. From ongoing work in our lab, we have observed that while one or two 1-min swirling stressors (spaced apart by 10 min; see **Figure 1A**) elicit behavioral hyperactivity, subjecting the larvae to three or four stressors instead results in diminished locomotion at levels similar to sham/unstressed controls (J.J. Rajeswari, G.N. Gilbert and M.M. Vijayan, *in prep*). Likewise, in the present study, repeated homotypic exposures to the swirling stressor no longer elevated *c-fos* following the 4^th^ instance of the stressor as compared to a single stressor (**Figure 1B**). This agrees with several lines of evidence from the mammalian literature showing an adaptation of the *c-fos* response to repeated stressors (34–36), validating the conserved role and use of this marker for habituation in larval zebrafish stress studies.

**Figure 9:**
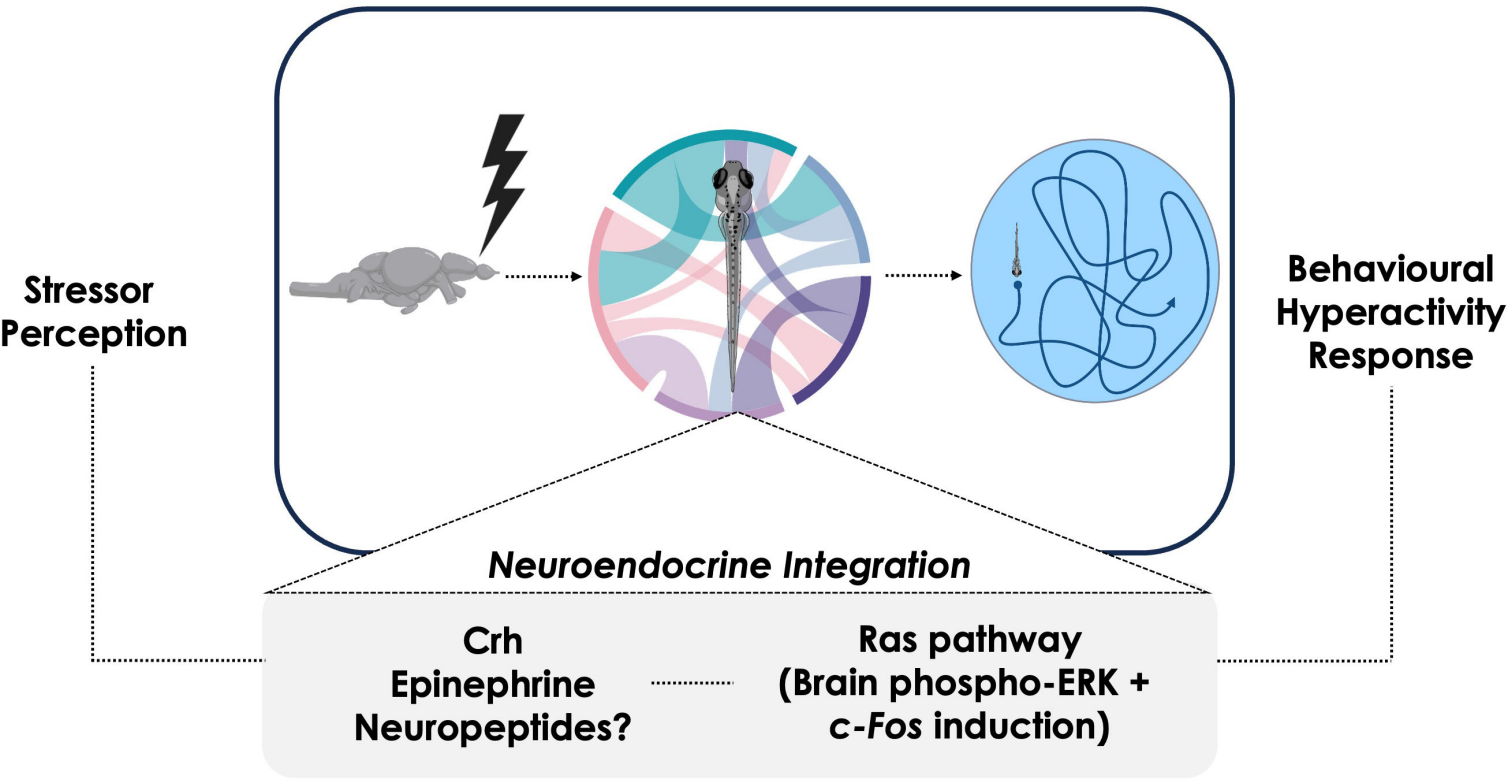
Summary diagram of proposed stress axis roles during the behavioral hyperactivity response in zebrafish larvae. Following stress perception, several neural and neuroendocrine mechanisms enact physiological changes by integrating the stress-specific stimulus. Many of these mechanisms impinge upon Ras-ERK signaling, with this cascade playing a central role in the activation of stress-related brain regions. Ultimately, the various neural and neuroendocrine components are integrated and together enact the coordinated behavioral hyperactivity at the organismal level.

In summary, our work identified common intracellular signaling mechanisms, including the Ras-Mapk pathway, utilized by both Crh and epinephrine in mediating the stress-related acute hyper locomotory activity in zebrafish larvae (**Figure 9**). This corresponded with rapid phosphorylation of Erk in brain regions of zebrafish associated with learning and stress responses in a Ras-dependent manner, likely driving the observed changes in behavioral outcomes. Although, both epinephrine and Crh elicited the rapid hyper locomotory response to an acute stress, the magnitude of response was greater with epinephrine, and not modulated by co-treatment with Crh, supporting a primary role for this stress hormone in the fight-or-flight response. However, our results indicate a key role of Crhr1 activation in modulating the epinephrine-induced locomotory response, and this may involve adrenoceptor regulation to increase hormonal responsiveness. We also show that the immediate early gene *c-fos*, a downstream target of the Mapk pathway, is modulated by epinephrine, but not Crh-Crhr1signaling, highlighting a stress-hormone-specific usage of this common intracellular effector in differing contexts. Furthermore, the *c-fos* gene is additionally responsive to physical swirling stressors and regulated by the Ras-Mapk pathway activation. However, despite the apparent shared usage of Mapk pathways by both Crh and epinephrine, downstream signaling appears to diverge past this point as reflected by the lack of effect on *c-fos* by Crh-Crhr1 signaling. Altogether, our results underscore a key role for the Crh-Crhr1 system in modulating the SNS-mediated fight-or-flight response but not the c-Fos response to stressor habituation in fish.

## Data Availability

The original contributions presented in the study are included in the article/**Supplementary Material**. Further inquiries can be directed to the corresponding author.

## Supplementary material

Supplementary Figure 1 Lack of changes in HPI transcript abundance following epinephrine or Crh treatment. 4dpf WT larvae were treated in 6 well plates with epinephrine **(A)** or Crh **(B)** and sampled at 1 h for analysis of transcript abundance related to hypothalamus-pituitary-interrenal (HPI) axis components (*gr*, *mr*, *pomca*, *pomcb*), with or without BAY-293 (10 μM) pre-treatment (2 h).
