# Supplementary figures and images for: Crhr1 and epinephrine utilize the central Ras-MAPK pathway in mediating the acute stress-related locomotor activity in zebrafish larvae
Source: Front Endocrinol (Lausanne). 2025 Sep 11;16:1650458. doi: 10.3389/fendo.2025.1650458 (PMC12460149; doi:10.3389/fendo.2025.1650458)

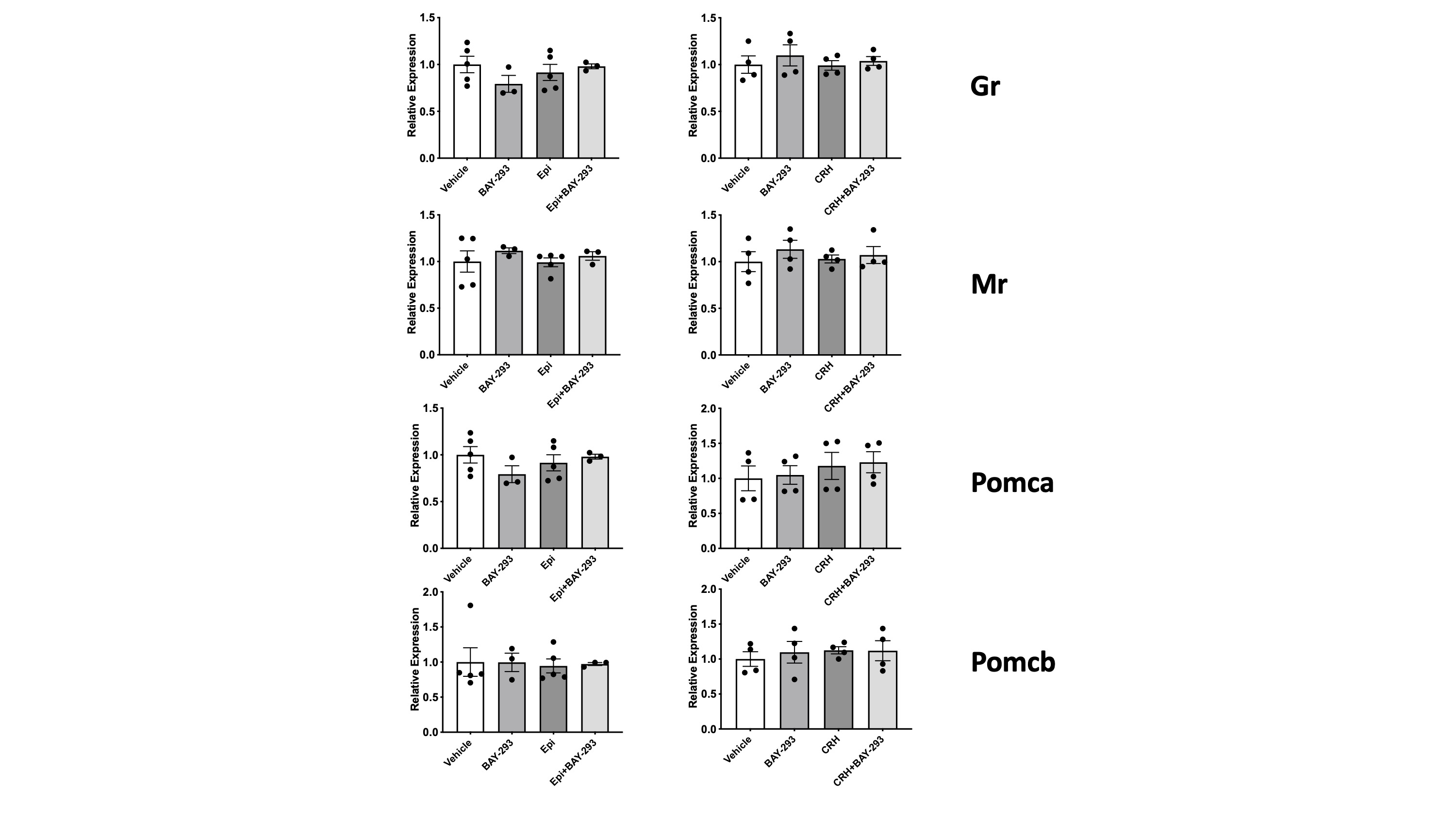

Supplement: Supplementary Figure 1 — Lack of changes in HPI transcript abundance following epinephrine or Crh treatment. 4dpf WT larvae were treated in 6 well plates with epinephrine (A) or Crh (B) and sampled at 1 h for analysis of transcript abundance related to hypothalamus-pituitary-interrenal (HPI) axis components (gr, mr, pomca, pomcb), with or without BAY-293 (10 μM) pre-treatment (2 h). [file Image1.jpeg]
